# A Potentiometric Indirect Uric Acid Sensor Based on ZnO Nanoflakes and Immobilized Uricase

**DOI:** 10.3390/s120302787

**Published:** 2012-03-01

**Authors:** Syed M. Usman Ali, Zafar Hussain Ibupoto, Muhammad Kashif, Uda Hashim, Magnus Willander

**Affiliations:** 1 Department of Science and Technology, Linköping University, Campus Norrköping, Norrkoping SE-60174, Sweden; E-Mails: zafar.hussain.ibupoto@liu.se (Z.H.I.); magwi@itn.liu.se (M.W.); 2 Department of Electronic Engineering, NED University of Engineering and Technology, Karachi 75270, Pakistan; 3 Nano Biochip Research Group, Institute of Nano Electronic Engineering (INEE), University Malaysia Perlis, Kangar, Perlis 01000, Malaysia; E-Mails: kashif_bme@yahoo.com (M.K.); uda@unimap.edu.my (U.H.)

**Keywords:** ZnO nanoflakes (ZnO-NFs), potentiometric nanosensor, uricase, Nafion^®^ membrane

## Abstract

In the present work zinc oxide nanoflakes (ZnO-NF) structures with a wall thickness around 50 to 100 nm were synthesized on a gold coated glass substrate using a low temperature hydrothermal method. The enzyme uricase was electrostatically immobilized in conjunction with Nafion membrane on the surface of well oriented ZnO-NFs, resulting in a sensitive, selective, stable and reproducible uric acid sensor. The electrochemical response of the ZnO-NF-based sensor *vs.* a Ag/AgCl reference electrode was found to be linear over a relatively wide logarithmic concentration range (500 nM to 1.5 mM). In addition, the ZnO-NF structures demonstrate vast surface area that allow high enzyme loading which results provided a higher sensitivity. The proposed ZnO-NF array-based sensor exhibited a high sensitivity of ∼66 mV/ decade in test electrolyte solutions of uric acid, with fast response time. The sensor response was unaffected by normal concentrations of common interferents such as ascorbic acid, glucose, and urea.

## 1. Introduction

Uric acid (UA) is the main end product of purine metabolism, and its excretion in urine is caused by purines that are produced in the catabolism of the dietary and endogenous nucleic acid. The production of excess uric acid may precipitate in the kidney and the lower extremities. One problem caused by the metabolism of the uric acid is gout [1]. Several epidemiological studies have suggested that the production of excess uric acid in human serum is also a risk factor for cardiovascular disease [2]. Thus, the detection of UA in human physiological fluids is necessary for the diagnosis of patients suffering from a range of disorders associated with altered purine metabolism. Recently, various uric acid biosensors have emerged from laboratories, because of the advantages of simple measurement, a short response time, high sensitivity, and high selectivity [3–7]. Most uric acid biosensors are based on amperometric principles [8–11]. The main problem in the practical application of many amperometric biosensors is that the electrode must be held at approximately 0.7 V [12]. The relatively high electrode potential enables other biological electroactive molecules to react on the surface of the electrode [13]. However, interferences can be reduced by preferring potentiometric configuration as described in our earlier investigations [14–17]. Recent advances in the biocompatible nanomaterials and biotechnology open a promising field toward the development of the nanostructured based electrochemical biosensing. Among the nanomaterials, zinc oxide (ZnO) is of special interest for biological sensing due to its many favorable properties like a wide direct band gap (3.37 eV) and large exciton binding energy (60 meV). In addition, ZnO has high ionic bonding (60%), and it dissolves very slowly at biological pH values.

Recently, a number of scientific investigations based on different ZnO nanostructures fabricated by various physical and chemical routes have been reported for sensing applications. These include nanowires/nanorods [18] nanotubes [19,20] combs [21,22] forks [23], fibers [24], flakes [25], composites [26], tetrapods [27], particles [28], flowers [29], sheet/disks [30], *etc.* Due to their unique advantages in combination with immobilized enzymes, these ZnO nanosensors offer some significant advantages owing to their small size and high surface area to volume ratios allowing larger signals, better catalysis and the more rapid movement of analyte through sensors, thus showing higher sensitivity and a lower limit of detection (LOD) as compared to those prepared from bulk ZnO devices. ZnO nanoflake (ZnO-NF) structures possess lots of interesting unique properties such as porous structures and large surface areas and there have been reports on the use of ZnO-NF structures as sensors with improved performance and higher sensitivity compared to ZnO nanorods/nanowires [25]. Moreover, ZnO has a high isoelectric point (IEP) of about 9.5, which should provide a positively charged substrate for immobilization of low IEP proteins or enzyme such as uricase (IEP ≈ 4.6) as described in our earlier investigations [31–34].

In this study, we have successfully demonstrated the potentiometric determination of uric acid with high electrochemical response by using a ZnO-NF-based sensor fabricated by a hydrothermal method. This method has many advantages such as being a low cost, simple, high yield, low temperature deposition process and also proves to be less hazardous compared to other methods. The high electrochemical response can be attributed to the unique structural properties of our sensor electrode like the high surface to volume ratios of ZnO-NFs, which can provide a favorable microenvironment for the immobilization of uricase enzyme and retain the good enzymatic activities which in turn enhances the sensitivity of sensor electrode for the analyte, as demonstrated by the detection of uric acid in the absence of a mediator.

## 2. Experimental

### 2.1. Reagents

Uricase (E.C. 1.7.3.3), 25 units/1.5 mg from *Arthrobacter globiformis*, uric acid (99.8% purity), β-d-glucose (99.5%), Nafion (1% in methanol), zinc nitrate hexahydrate and hexamethylenetetramine were purchased from Sigma Aldrich. Phosphate Buffer, 10 mM solution (PBS) was prepared from Na_2_HPO_4_ and KH_2_PO_4_ (Sigma Aldrich) with sodium chloride in 0.135 mM and the pH was adjusted to 7.4. A stock solution of 10 mM uric acid was prepared in PBS, and stored at 4 °C. The low concentration standard solutions of the uric acid were freshly prepared before the measurements. All chemicals used (Sigma, Aldrich) were of analytical reagent grade.

### 2.2. Fabrication ZnO-NPs Arrays Based Sensor Electrode

**Figure 1.**
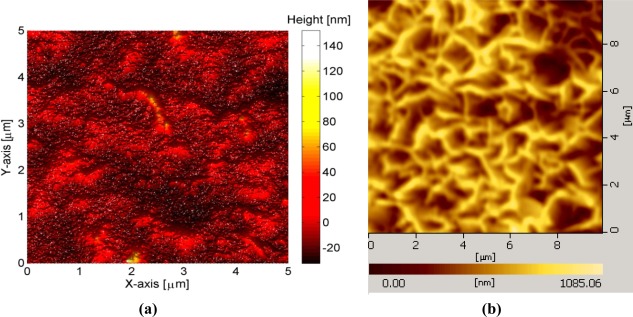
(**a**) AFM image of deposited gold thin film on glass substrate showing a flat surface with a surface roughness of Ra = 10 nm and (**b**) AFM image of grown ZnO-NFs arrays.

To fabricate the sensor electrodes, glass substrates were used after being cleaned with acetone and de-ionized water then we affixed the glass substrate on a flat support inside the vacuum chamber of an evaporation system (Evaporator Satis CR725). In the first step, a thin film of titanium (Ti) with 20 nm thickness was evaporated as an adhesive layer then gold (Au) thin film with 100 nm thickness was evaporated. An AFM image showing the surface roughness of deposited gold films is shown in Figure 1(a).

In the second step, a small part of the gold coated glass was covered with plastic in order to make a contact area, and then the 10 nm of Aluminum (Al) thin film was deposited on the remaining selective portion of the gold coated substrate. To fabricate the ZnO-NFs on the prepared electrode, a low temperature hydrothermal approach was adopted [35]. First the seed solution containing zinc acetate in methanol was spun coated for 30 seconds at 3,000 rpm and then annealed in a preheated oven at 200 °C for five minutes. Then the electrodes were placed in an aqueous solution that was prepared in deionized water (150 mL) with 0.025 M zinc nitrate hexahydrate [(Zn (NO)_3_)_2·_6H_2_O)] and 0.025 M hexamethylenetetramine [C_6_H_12_N_4_] that was kept in preheated an oven at 90 °C for 2–4 hours. After the growth process, the fabricated ZnO-NFs were cleaned in de-ionized water and dried at room temperature. A typical AFM image of ZnO-NPs arrays grown on the gold coated plastic electrode using this procedure are shown in Figure 1(b).

**Figure 2.**
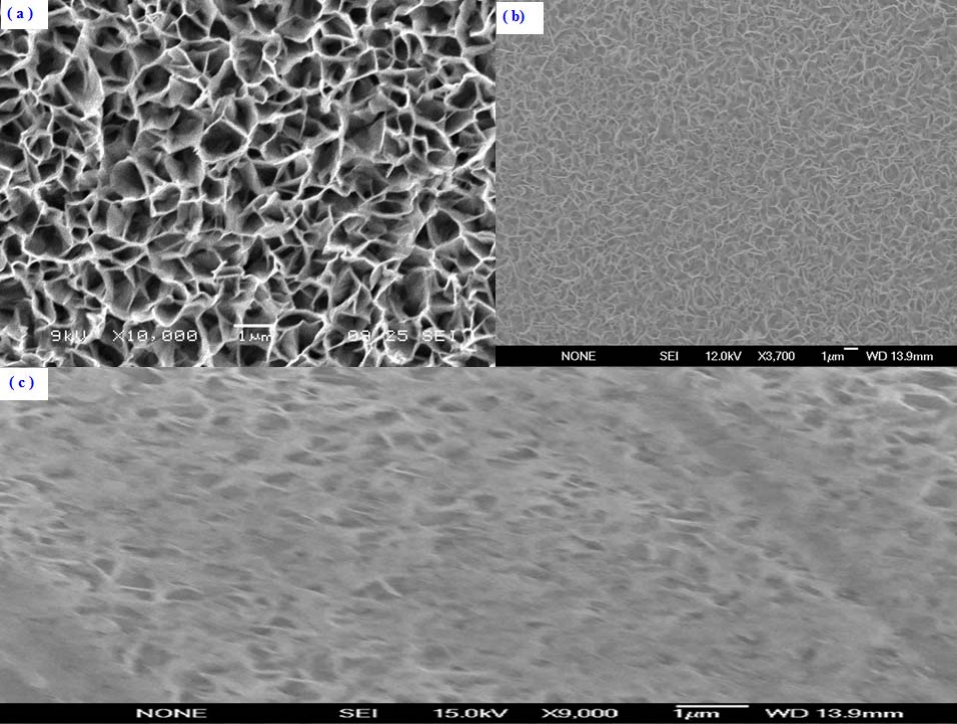
A typical SEM images of ZnO-NFs arrays grown on gold coated glass substrate using low temperature chemical growth. The figure showing (**a**) the ZnO-NFs arrays as fabricated; (**b**) with immobilized uricase and (**c**) the same sensor electrode after measurements.

The morphological and structural studies were performed by using Scanning Electron Microscopy (SEM). The SEM images of the ZnO-NFs with as fabricated, after enzymes immobilization and after measurements are shown in Figure 2(a–c).

It can be clearly seen that the wall thickness of the grown ZnO-NFs are 50–100 nm in diameter with uniform density. These ZnO-NFs were well oriented on the surface of the electrodes. The morphological and structural characteristics of the fabricated ZnO-NFs arrays can be controlled by adjusting the growth parameters.

### 2.3. Enzymes Immobilization on ZnO-NFs

To immobilize the uricase enzyme on the fabricated ZnO-NFs, first we have prepared an uricase solution in 10 mM PBS pH 7.4. Uricase was electrostatically immobilized by dipping the ZnO-NF-based electrode into the enzyme solution for 15 minutes at room temperature and then for drying, it was left in air for 60 min. After drying, Nafion solution (1% in methanol, 5 μL) was applied onto the electrode surface to prevent possible enzyme leakage and eliminate foreign interferences. All enzyme electrodes were stored in dry conditions at 4 °C when not in use. After completing all these steps, the prepared sensors were checked potentiometrically in uric acid solutions with an Ag/AgCl reference electrode purchased from Metrohm. A pH meter (Model 744, Metrohm) was used to measure the potentiometric output voltage of the ZnO-NFs based sensors presented here. For the time response measurements, a model 363A potentiostat/galvanostat (EG & G, Las Vegas, NV, USA) was used. Atomic force microscopy images were acquired using a Dimension 3100 Scanning Probe Microscope (Digital Instruments) in tapping mode with Si cantilevers.

## 3. Results and Discussion

### 3.1. The Electrochemical Response of ZnO-NFs Sensors

The electrochemical measurements were carried out using a two-electrode configuration consisting of the ZnO-NF-based sensor as the working electrode and an Ag/AgCl one as a reference electrode. The electrochemical response of the ZnO-NFs sensor *versus* an Ag/AgCl reference electrode was measured at room temperature (23 ± 2) °C. The sensor as fabricated is sensitive to the concentration changes of uric acid in PBS. An electrochemical response from ZnO-NFs sensor in the 100 μM uric acid solution was observed around 200 mV. The response stayed around 200 mV regardless of the analyte solution volume. During all experiments the ZnO-NFs sensor followed the Nernst’s expression:

E=E0−0.05916 V/n log [Reduced]/[Oxidized]

**Figure 3.**
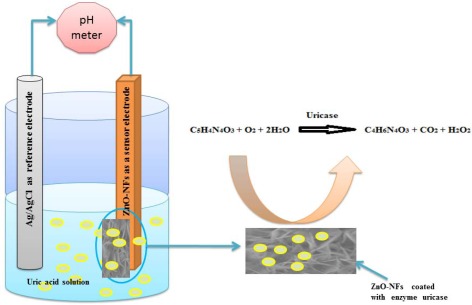
Schematic diagram of the uric acid sensing setup using ZnO-NFs coated with uricase as working electrode showing the possible electrochemical reaction near the working electrode.

It is very important to note that ZnO-NFs are relatively stable around a neutral pH of 7.4 and this gives these sensors much more bio-compatibility in biological fluids and species since the pH of most biological fluids is around 7.4. The sensing mechanism of most electrochemical uric acid sensors is based on an enzymatic reaction catalyzed by uricase as described in Figure 3.

When uric acid is oxidized in the presence of uricase it is turned into allantoin along with carbon dioxide and hydrogen peroxide. Due to the presence of water (H-OH), it is a high probability that allantoin will accept a proton from (H-OH) converting it to allantoinium ion, which in turn will interact with the ZnO-NFs and produce a potential change at the electrode. As the concentration of ions changes in surrounding the ZnO-NFs and the electrode potential will change [36]. The potentiometric responses of the sensor electrodes were studied in uric acid solutions made in buffer (PBS pH 7.4) with concentration ranging from 0.5 μM to 1,500 μM. During the measurements it was observed that the carbon dioxide produced does not affect the stability of ZnO-NFs as shown in SEM image of Figure 2(c) and we did not observe any substantial change in pH of the buffer solution (PBS).

**Figure 4.**
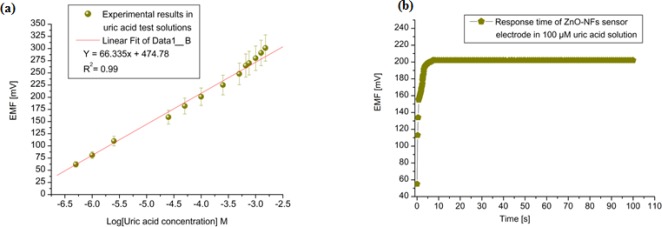
(**a**) Calibration curve for the ZnO-NFs based uric acid sensor and (**b**) Time response of the ZnO-NFs based uric acid sensor in 100 μM uric acid solution.

The tested sensor configuration showed large dynamic ranges with an output response (emf) that was linear *vs.* the logarithmic concentrations of the uric acid with sensitivity around 66 mV/decade as shown in Figure 4(a). A very fast response time was noted over the whole concentration range with 95 % of the steady state voltage achieved within 8 s, as shown in Figure 4(b).

### 3.2. Reproducibility, Measuring Range and Detection Limit of the ZnO-NFs Based Sensor

**Figure 5.**
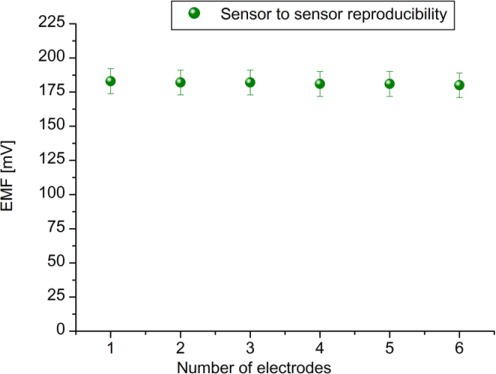
The sensor to sensor reproducibility of six (n = 6) ZnO-NFs /uricase/Nafion electrodes in 100 μM uric acid solution.

To evaluate the performances of the proposed sensor, we have checked the parameters like reproducibility, measuring range, detection limit, response time and selectivity, *etc.* The reproducibility is an important characteristic for the performance evaluation of a sensor. To evaluate reproducibility and long term stability of the proposed ZnO-NFs based sensors, we independently fabricated six sensor electrodes under the same conditions; the relative standard deviation of the fabricated sensor electrodes in standard uric acid solutions was less than 5%. The sensor to sensor reproducibility in 100 μM uric acid solution is shown in Figure 5.

The measuring range of the proposed sensor was obtained from the linear part of the calibration graph as shown in Figure 4(a). The applicable measuring range of the proposed sensor is between 0.5 μM to 1500 μM. By extrapolating the linear parts of the calibration curve, the detection limit of ZnO-NFs based sensor electrode could be calculated. In the present work the detection limit of the sensor was 0.5 μM which was calculated by the extrapolating of the two segments of the calibration curve shown in Figure 4(a). This sensor electrode had been periodically used and stored at 4 °C for more than three weeks; it retained up to 80% of its original activity and still showed a good response to uric acid. It has been observed that ZnO-NFs provide a suitable environment for immobilization of high number of enzyme molecules with firm binding due to entangled structure of nanoflakes, which in result showed a fast response and high sensitivity towards the uric acid detection. Thus, our proposed uric acid sensor based on the ZnO nanoflake structure offers a promising enzyme immobilization platform for the fabrication of sensors which can be used for real sample applications in bioanalysis. During the fabrication of uric acid sensor, the loading of uricase enzyme is highly crucial and especially when the sensor is treated in real sample analysis. The sensor based on nanoflakes exhibited a minor loss of enzyme molecules during the experiments. Thus, it could be concluded that sensor may behave in a similar fashion in a real sample because it was operated under the same conditions as for real samples. The morphology of the functionalized ZnO-NFs based sensors electrode was checked by scanning electron microscopy (SEM) after measurements, as shown in Figure 1(c).

### 3.3. Selectivity of the ZnO-NFs Arrays Based Sensor

**Figure 6.**
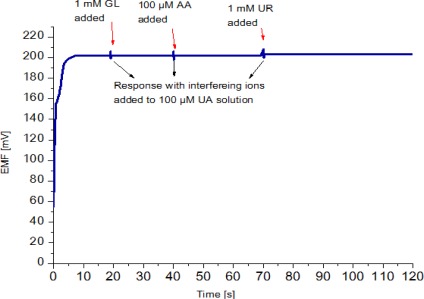
Effect of potentially interfering substances on sensor response (emf) upon adding 1 mM glucose (GL), 100 μM ascorbic acid (AA) and urea (UR) into 100 μM uric acid solution.

Selectivity is the most important characteristic which describes the specificity towards the target ion in the presence of other ions (interfering ions). There are different methods to determine selectivity of the potentiometric sensors [37]. These methods are the separate solution method, the mixed solution method, the matched potential method, and the unbiased selective coefficients. Instead of using the above mentioned methods, we checked the selectivity and stability of the sensor by output response curve. The possible interferences present in blood that normally interfere with an amperometric uric acid biosensor include ascorbic acid (AA) urea (UR) and glucose (GL) [38]. Hence, ascorbic acid, urea and glucose were selected to affirm the selectivity of the potentiometric uric acid sensor. In the present work, upon addition of 1 mM glucose, 100 μM ascorbic acid and 1mM urea solutions in a 100 μM uric acid solution the signal changed only slightly, which indicates a good selectivity, as shown in Figure 6.

This was repeated several times on new, independently prepared sensors and continued to show negligible signal response to interferences. In practical measurements, however these changes in sensor response can be neglected.

## 4. Conclusions

In conclusion, we have successfully demonstrated a simple fabrication procedure for a highly sensitive electrochemical uric acid sensor based on ZnO nano-flake-based structures. The proposed electrochemical nanosensor demonstrates immense surface area to volume ratios which provide a suitable microenvironment for enzyme loading because of its porosity that allows for very good sensitivity as compared to other ZnO nanostructures as shown in Table 1, portability and small size.

The uricase sensor retained its enzymatic activity due to strong electrostatic interaction between zinc oxide and uricase. Moreover, the developed ZnO-nanoflake-based nanosensor showed excellent performance regarding sensitivity, stability, selectivity, reproducibility and resistance to interference when the sensor was exposed to uric acid test solutions. These results revealed that electrochemical sensors based on ZnO nanoflakes have the potential to perform measurements biologically relevant to on-spot clinical diagnosis. They are also convenient to assemble into portable chip based sensing devices suitable for unskilled users.

## Figures and Tables

**Table 1.** Comparison of some uric acid sensors based on different ZnO nanostructures.

**Transducer**	**Matrix**	**Sensitivity**	**Response time**	**Shelf life**	**Range**	**Reproducibility**	**Reference**
**Potentiometric**	ZnO nanowires	29 mV/decade	6–9 s	12 weeks	1 μM–1,000 μM	20 times	[31]
**Potentiometric**	ZnO nanotubes	68 mV/decade	8 s	12 weeks	0.5 μM–1,500 μM	20 times	[32]
**Amperometric**	ZnO nanorods	-------	-------	20 days	5 μM–1 mM	10 times	[33]
**Amperometric**	ZnO nanoparticles	393mA cm^−2^M^−1^	∼8 s	12 weeks	5 μM–1 mM	---------	[34]
**Potentiometric**	ZnO nanoflakes	∼66 mV/decade	∼8 s	12 weeks	500 nM–1.5 mM	20 times	[present]
